# Analysis of Milestone-based End-of-rotation Evaluations for Ten Residents Completing a Three-year Anesthesiology Residency

**DOI:** 10.7759/cureus.3200

**Published:** 2018-08-24

**Authors:** Chloe M Chemtob, Pedro Tanaka, Martin Keil, Alex Macario

**Affiliations:** 1 Anesthesiology, Stanford University, Palo Alto, USA; 2 Anesthesiology, Perioperative and Pain Medicine, Stanford University School of Medicine, Stanford, USA; 3 Graduate School of Education, Stanford University, Stanford, USA

**Keywords:** residency programs, anesthesiology, straight-line scoring, perioperative and pain medicine, medical education, milestones, resident evaluation, sub-competency, graduate medical education

## Abstract

Introduction

Faculty are required to assess the development of residents using educational milestones. This descriptive study examined the end-of-rotation milestone-based evaluations of anesthesiology residents by rotation faculty directors. The goals were to measure: (1) how many of the 25 Accreditation Council for Graduate Medical Education (ACGME) anesthesiology subcompetency milestones were included in each of the residency’s rotations evaluations, (2) the percentage of evaluations sent to the rotation director that were actually completed by the director, (3) the length of time between the end of the residents' rotations and completion of the evaluations, (4) the frequency of straight line scoring, defined as the resident receiving the same milestone level score for all subcompetencies on the evaluation, and (5) how often a resident received a score below a Level 4 in at least one subcompetency in the three months prior to graduating.

Methods

In 2013, the directors for each the 24 anesthesia rotations in the Stanford University School of Medicine Anesthesiology Residency Program created new milestone-based evaluations to be used at the end of rotations to evaluate residents. The directors selected the subcompetencies from the list released by the ACGME that were most appropriate for their rotation. End-of-rotation evaluations for the post-graduate year (PGY)-2 to PGY-4 from July 1, 2014 to June 30, 2017 were retrospectively analyzed for a sample of 10 residents randomly selected from 22 residents in the graduating class.

Results

The mean number of subcompetencies evaluated by each of the 24 rotations in the residency equaled 17.88 (standard deviation (SD): 3.39, range 10–24, median 18.5) from the available possible total of 25 subcompetencies. Three subcompetencies (medical knowledge, communication with patients and families, and coordination of patient care within the healthcare system) were included in the evaluation instruments of all 24 rotations. The three least frequently listed subcompetencies were: “acute, chronic, and cancer-related pain consultation/management” (25% of rotations had this on the end-of-rotation evaluation), “triage and management of critically ill patient in non-operative setting” (33%), and “education of patient, families, students, residents, and others” (38%). Overall, 418 end-of-rotation evaluations were issued and 341 (82%) completed, with 63% completed within one month, 22% between month one and two, and 15% after two months. The frequency of straight line scoring varied, from never occurring (0%) in three rotations to always occurring (100%) in two rotations, with an overall average of 51% (SD: 33%). Sixty-one percent of straight line scoring corresponded to the residents’ postgraduate year whereby, for example, a post-graduate year two resident received an ACGME Level 2 proficiency for all subcompetencies. Thirty-one percent of the straight line scoring was higher than the resident’s year of training (e.g., a PGY-2 received Level 3 or higher for all the subcompetencies). The remaining 7% of straight line scoring was below the expected level for the year of training. Three of seven residents had at least one subcompetency rated as below a Level 4 on one of the evaluations during the three months prior to finishing residency.

Conclusion

Formal analysis of a residency program’s end-of-rotation milestone evaluations may uncover opportunities to improve competency-based evaluations.

## Introduction

The Accreditation Council for Graduate Medical Education (ACGME) introduced the Next Accreditation System in 2013 [1]. This outcomes-based model of residency relies on trainees showing competency in a variety of skills, attitudes, and knowledge to track their progress with the ultimate goal of a smooth transition to independent practice. Assessments in graduate medical education rely heavily on workplace-based observations by faculty [2,3].

The Next Accreditation System incorporated the six ACGME core competencies that apply to all graduate medical education: Patient Care, Medical Knowledge, Professionalism, Interpersonal and Communications Skills, Practice-based Learning and Improvement, and Systems-based Practice. Each specialty then independently created a specific set of subcompetencies for each of the six core competencies that would be used by that specialty’s residency programs to develop curriculum and assessment.

For anesthesiology, a total of 25 subcompetencies exist, ranging from one subcompetency for the Medical Knowledge core competency to 10 subcompetencies for the Patient Care competency [4]. For example, the Interpersonal and Communications Skills core competency for anesthesiology has three subcompetencies: communication with patients and families, communication with other professionals, and team and leadership skills. Residents are meant to be evaluated based on their performance in the individual subcompetencies.

Each subcompetency then has a set of milestones, from less to more advanced, which define the target behaviors for performance. The milestones are arranged into numbered proficiency levels from Level 1 whereby the resident “demonstrates milestones expected of a resident who has completed one post-graduate residency year” to Level 5 whereby the resident “has advanced beyond performance targets defined for residency, and is demonstrating “aspirational” goals such as the performance of someone who has already been in independent practice for several years. Only a few exceptional residents should reach this Level 5 for any of the subcompetencies.” Anesthesiology milestones, as assessed by the faculty, have a positive linear relationship with post-graduate year level [5].

This study aimed to examine the end-of-rotation milestone-based evaluations of residents as completed by faculty for academic years 2014-2017. The motivation for the study was to better understand the assessment data being collected to make improvements in the evaluation of residents. More specifically, some residents reported they could gain more insight about their professional development if the end-of-rotation evaluations had more useful feedback information. Also, the department’s Clinical Competence Committee uses the end-of-rotation evaluation data to make decisions on milestone levels submitted by the residency to the ACGME via the online Accreditation Data System account.

The goals were to measure: (1) how many of the 25 ACGME anesthesiology subcompetencies were included in each of the residency’s rotations evaluations, (2) the percentage of evaluations sent to the rotation director that were actually completed by the director, (3) the length of time between the end of the resident’s rotation and completion of the evaluation (because delays may be associated with a decreased ability to detect differences in resident performance [6]), (4) the frequency of straight line scoring, defined as the resident receiving the same milestone level score for all subcompetencies on the evaluation, and (5) how often did a resident received a score below a Level 4 in at least one subcompetency in the three months prior to graduating.

## Materials and methods

After approval from the Stanford University Humans Subjects Committee, data from end-of-rotation milestone-based evaluations for post-graduate year (PGY) 2/Clinical Anesthesia 1 to PGY-4/Clinical Anesthesia 3 from July 1, 2014 to June 30, 2017 were retrospectively analyzed for a sample of 10 randomly selected (via a random number generator) from the 22 graduating residents.

After the ACGME published the 25 anesthesiology subcompetencies in 2013, and after all the rotation directors in the Stanford Anesthesiology residency received an hour-long, small group education session with the residency program director on the rationale for the milestones, each rotation director created new end-of-rotation evaluation instruments by selecting the subcompetencies that were most appropriate for their rotation.

These newly created evaluations were launched for all rotations in the residency on July 1, 2014.

After the resident completed a rotation, the end-of-rotation evaluation form was sent to the faculty rotation director via the web-based Residency Management System (MedHub, Minneapolis, MN). If the evaluation was not completed within a month, a reminder was sent, followed by another reminder a month later. There was no specific repercussion to the faculty for delay or failure to submit.

Each subcompetency of the end-of-rotation evaluation was rated on a 10-point milestone scale, from 0.5 (not yet achieved Level 1) 1, 1.5, 2, 2.5, 3, 3.5, 4, 4.5 or 5, with the 0.5 indicating performance between the level below and the level above.

Rotation evaluation forms included space for general qualitative comments, but these were excluded from this study.

## Results

Data from the 10 randomly selected residents were analyzed with a total of 418 end-of-rotation milestone-based evaluations issued between 7/1/2014 and 6/30/2017. Three of the residents had medical leaves of absence during residency which extended the finish date of the residency past July 1, 2018 and affected the number of available rotation evaluation data.

1. How many of the 25 anesthesiology subcompetencies were included in each of the end-of-rotation evaluations?

Of the 25 total available anesthesiology subcompetencies, the mean number of subcompetencies evaluated by each rotation equaled 17.88 (standard deviation (SD): 3.39, range 10–24, median: 18.5).

Three subcompetencies (medical knowledge, communication with patients and families, and coordination of patient care within the health-care system) were assessed in all 24 rotations (Table 1).

**Table 1:** Subcompetencies included in the end-of-rotation evaluation of the resident for the 24 clinical rotations (1 = Yes included, 0 = Not included). OR 1: Hospital #1 general OR OR 2: Hospital #2 general OR Pre-Op 1: Hospital 1 Pre-Op OR 3: Hospital #3 general OR Chief 3: Hospital 3 Chief Resident OR: Operating room; Ob: Obstetrics; MICU: Medical intensive care unit; PACU: Post anesthesia care unit; SICU: Surgical intensive care unit; CTICU: Cardiothoracic intensive care unit; Peds CV: Pediatric cardiovascular; ENT: Otolaryngology; ABA: American Board of Anesthesiology; QI: Quality improvement.

		Name of Rotation																							
	Percentage of 24 Rotations that Evaluated	OR 1	OR 2	MICU	Acute Pain	Bariatrics	Pre-Op 1	Neuro	Ob	Heart	Pediatrics	Liver	Thoracic	Spine	Regional	OR3	PACU	SICU	Abdomen	CTICU	Chief 3	Peds CV	ENT	Ortho Trauma	Chronic Pain
Patient Care																									
1. Preanesthetic evaluation, assessment, and preparation	92%	1	1	0	1	1	1	1	1	1	1	1	1	1	1	1	1	1	1	0	1	1	1	1	1
2. Anesthetic plan and conduct	79%	1	1	0	1	1	0	1	1	1	1	1	1	1	1	1	1	0	1	0	1	1	1	0	1
3. Periprocedural pain management	58%	1	1	0	0	1	1	0	0	0	1	1	0	1	0	1	1	0	0	1	1	0	1	1	1
4. Management of perianesthetic complications	67%	1	1	1	0	0	1	0	1	0	1	0	1	1	0	1	1	1	0	1	1	1	1	1	0
5. Crisis management	67%	1	1	1	0	0	0	0	1	1	1	1	0	1	0	1	1	1	0	1	1	1	1	1	0
6. Triage and management of critically ill patient in non-operative setting	33%	0	1	1	0	0	0	0	1	0	1	0	0	1	0	0	0	1	0	1	0	0	0	1	0
7. Acute, chronic, and cancer-related pain consultation/management	25%	0	0	0	1	0	0	0	1	0	0	0	0	1	0	0	0	0	1	0	0	0	0	1	1
8. Technical skills: airway management	75%	1	1	1	0	1	0	1	1	1	1	1	0	1	1	1	0	1	0	1	1	1	1	1	0
9. Technical skills: use/interpretation of monitoring and equipment	75%	1	1	1	0	1	0	1	1	1	1	1	0	1	1	1	0	1	1	1	1	1	1	0	0
10. Technical skills: regional anesthesia	38%	1	1	0	0	0	0	0	0	1	1	0	0	0	1	1	0	0	0	1	1	0	1	0	0
Medical Knowledge																									
1. Knowledge (ABA)	100%	1	1	1	1	1	1	1	1	1	1	1	1	1	1	1	1	1	1	1	1	1	1		1
Professionalism																									
1. Responsibility to patients, families, and society	96%	1	1	1	1	1	1	1	1	1	0	1	1	1	1	1	1	1	1	1	1	1	1	1	1
2. Honesty, integrity, and ethical behavior	79%	1	1	1	1	1	0	1	1	0	1	0	1	1	0	1	1	1	0	1	1	1	1	1	1
3. Commitment to institution, department, and colleagues	79%	1	1	1	1	1	0	1	0	1	1	0	1	1	1	1	1	1	1	0	1	1	1	0	1
4. Receiving and giving feedback	50%	1	1	1	0	0	0	0	1	0	0	0	0	1	1	0	0	1	1	1	0	1	1	1	0
5. Responsibility for personal emotional, physical, and mental health	45%	0	1	0	0	0	0	0	1	0	0	1	0	0	0	1	0	1		1	1	1	1	1	
Interpersonal and Communications Skills																									
1. Communication with patients and families	100%	1	1	1	1	1	1	1	1	1	1	1	1	1	1	1	1	1	1	1	1	1	1	1	1
2. Communication with other professionals	96%	1	1	1	1	1	1	1	1	1	1	1	1	1	1	1	1	1	0	1	1	1	1	1	1
3. Team and leadership skills	83%	1	1	1	1	1	0	1	1	1	0	1	0	1	0	1	1	1	1	1	1	1	1	1	1
Practice-based Learning and Improvement																									
1. Incorporation of QI & patient safety initiatives into personal practice	79%	1	1	1	1	1	1	1	1	1	1	0	0	0	1	0	1	1	0	1	1	1	1	1	1
2. Analysis of practice to identify areas in need of improvement	92%	0	1	1	1	1	1	1	1	1	1	1	1	1	0	1	1	1	1	1	1	1	1	1	1
3. Self-directed learning	71%	1	1	0	0	0	0	0	1	0	1	1	1	1	1	1	1	1	1	0	1	1	1	1	1
4. Education of patient, families, students, residents, and others	38%	0	0	1	0	0	0	0	0	0	0	0	0	1	1	1	0	1	0	1	1	0	1	1	0
Systems-based Practice																									
1. Coordination of patient care within the health-care system	100%	1	1	1	1	1	1	1	1	1	1	1	1	1	1	1	1	1	1	1	1	1	1	1	1
2. Patient safety and quality improvement	79%	1	1	1	1	1	0	1	1	1	1	0	1	0	1	1	1	1	1	1	0	0	1	1	1
Number of subcompetencies evaluated		20	23	18	14	16	10	15	21	16	19	15	13	21	16	21	17	21	14	20	21	19	23	20	16

In contrast, the three least frequently assessed subcompetencies were: “acute, chronic, and cancer-related pain consultation/management” (25% of rotations had this listed on the end-of-rotation evaluation form of the resident), “triage and management of critically ill patient in non-operative setting” (33%), and “education of patient, families, students, residents, and others” (33%).

2. The percentage of evaluations sent to the rotation director that were actually completed by the director.

Overall, 82% of end-of-rotation evaluations issued to faculty were completed (Table 2).

**Table 2:** Percentage of end-of-rotation evaluations that were completed by rotation director.

Resident	Rotation Evaluations Completed	Number of Rotations	Percentage of Evaluations/Number of Rotations
1	42	46	91%
2	41	42	98%
3	42	46	91%
4	27	30	90%
5	25	32	78%
6	34	43	79%
7	33	46	72%
8	32	45	71%
9	35	47	74%
10	30	41	73%
TOTAL	341	418	82%

The total number of rotations (and evaluations completed) varied from resident to resident. This occurred because each resident had their own unique combination of rotations in their residency experience. Although there are 13 four-week rotation blocks per academic year, some of the four-week blocks have two different two-week rotations also affecting the number of rotations. Three residents had leaves of absences also impacting the number of rotations during the study period.

3. The length of time between the end of the resident’s rotation and completion of evaluation by the faculty rotation director.

Of the 341 milestone-based evaluations submitted for the 10 residents, 63% were completed within one month, 22% between month one and two, and 15% after two months (Table 3).

**Table 3:** The majority of evaluations were completed with one month of rotation end.

Resident	Percentage of Evaluations Completed within 1 Month	Percentage of Evaluations Completed between 1-2 Months	Percentage of Evaluations Completed after 2 Months
1	52%	29%	19%
2	56%	32%	12%
3	69%	17%	14%
4	74%	19%	7%
5	72%	16%	12%
6	62%	15%	24%
7	79%	12%	9%
8	63%	19%	19%
9	49%	34%	17%
10	60%	23%	17%
TOTAL	63%	22%	15%

4. How often was the resident assigned the same milestone level for all subcompetencies listed on the end-of-rotation evaluation?

Sixty-one percent of all evaluations had the same level score for all the subcompetencies on the end-of-rotation evaluation (Table 4).

**Table 4:** Percentage of rotation evaluations that had same level scoring for all subcompetencies listed on the evaluation.

Resident	Percentage of Evaluations with the Same Level Score for All Subcompetencies	Total Number of Evaluations	Percentage of Evaluations with the Same Level Score for All Subcompetencies
1	28	42	67%
2	27	41	68%
3	24	42	57%
4	16	27	59%
5	13	25	52%
6	22	34	65%
7	20	33	61%
8	18	32	56%
9	23	35	66%
10	17	30	53%
TOTAL	208	341	61%

The frequency of straight line scoring varied by rotation, from 0% in three rotations to 100% in two rotations with an overall mean average of 52% (SD: 33%) (Table 5).

**Table 5:** The frequency of straight line scoring varied by rotation. SLS: Straight line scoring; PGY: Post-graduate year; PACU: Post anesthesia care unit; OR: Operating room; ENT: Otolaryngology; VA OR: Veterans affairs hospital operating room; CTICU: Cardiothoracic intensive care unit; Peds CV: Pediatric cardiovascular; MICU: Medical intensive care unit; SICU: Surgical intensive care unit; SD: Standard deviation.

Rotation	Percentage of Evaluations with Straight Line Scoring	SLS below Corresponding PGY Level	SLS at Corresponding PGY Level	SLS above Corresponding PGY Level
Chronic Pain	100%	29%	57%	14%
PACU	100%	0%	83%	17%
OR 2	96%	1%	80%	18%
ENT	86%	0%	67%	33%
Chief 3	83%	0%	75%	25%
Spine	80%	13%	50%	38%
VA OR	79%	0%	14%	86%
Liver	75%	0%	100%	0%
Neuro1	67%	50%	0%	50%
Regional	60%	0%	50%	50%
OR 3	58%	0%	86%	14%
Acute Pain	56%	0%	80%	20%
CTICU	50%	0%	50%	50%
Pediatric	45%	0%	100%	0%
Thoracic	33%	0%	67%	33%
Peds CV	27%	0%	100%	0%
MICU	22%	50%	25%	25%
Obstetrics	21%	0%	25%	75%
Abdomen	20%	0%	100%	0%
VA Pre-Op	18%	0%	0%	100%
Bariatrics	0%			
Heart	0%			
SICU	0%			
Mean	51%	7%	61%	32%
SD	33%	16%	33%	29%

Sixty-two percent of SLS corresponded to the resident’s postgraduate year, 31% of the time the SLS was higher than the resident’s year of training, and the remaining 7% of the time was below the expected level for the year of training.

5. How commonly did a senior resident receive a score below a Level 4 in at least one subcompetency in the three months before graduating?

Three of the seven residents (the other three residents finished after the end of the study period due to leaves) had at least one subcompetency rated as below a Level 4 on one of the rotation evaluations during the three months prior to the completion of the residency program (Table 6).

**Table 6:** Frequency of a resident receiving a score below Level 4 on a subcompetency during the last three months of residency. *These residents finished residency after the end of the end period due to leaves of absence.

Resident	Number of Subcompetencies Rated below Level 4	Total Number of Subcompetencies Evaluated
1	0	24
2	10	52
3	*	*
4	*	*
5	*	*
6	0	67
7	0	41
8	0	56
9	7	59
10	1	43

## Discussion

Little is known about how anesthesiology residents are evaluated using milestones during rotations in a residency. This study found that faculty directors of 24 different clinical rotations at a large anesthesiology residency choose to include an average of 18 of the 25 available ACGME defined subcompetencies in the end-of-rotation evaluations. This indicates that most competencies can be evaluated several times during residency.

Whereas, three subcompetencies (medical knowledge, communication with patients and families, and coordination of patient care within the healthcare system) were assessed in all 24 rotations, “acute, chronic, and cancer-related pain consultation/management”, “triage and management of critically ill patient in non-operative setting”, and “education of patient, families, students, residents, and others” were evaluated in less than a third of the rotations. Different rotations have different resident duties and activities thereby defining what can be assessed during any rotation. For example, it may not be feasible to evaluate “acute, chronic, and cancer-related pain consultation/management” during some rotations.

The frequency of straight line scoring whereby the same score on the 10-point milestone scale is given to the resident in all of the subcompetencies varied by rotation, with an overall average of 51% (SD: 33%). This ranged from 0% in three different rotations (no resident on that rotation ever received straight line scoring) to 100% (every resident on that rotation received straight line scoring) in two rotations. When straight line scoring did occur, 61% corresponded directly to the resident’s postgraduate year. The milestone-based end-of-rotation evaluations may not be measuring the knowledge or ability possessed by the particular resident, but rather indicate where along the training sequence a resident falls.

In contrast, the results of this analysis show that 32% of the straight line scoring was higher than the resident’s year of training, and the remaining 7% of the time was below the expected level for the year of training.

Although milestone-based evaluations assume that each subcompetency is assessed independently with individualized assessments for each resident, a national study of milestones submitted to the ACGME by Family Medicine Residencies also found that residents are being rated in a stable manner as they progress through residency [7]. A separate study of all Emergency Medicine programs found that approximately 5% of the more than 6,000 residents had straight line scoring as reported to the ACGME. The data analyzed in both studies are different from this study because they analyzed data submitted to the ACGME every six months, instead of the individual end-of-rotation evaluations as was performed in this study.

In general, there are two domains for threats to the validity of assessments. The first is construct underrepresentation whereby there are not enough observations by the attending supervising resident. The second threat to validity is construct-irrelevant variance often primarily due to systematic rater error. Although raters are the major source of measurement error for these types of observational assessments, construct-irrelevant variance is associated with systematic rater error, such as rater severity or leniency error, central tendency error (rating in the center of the rating scale) and restriction of range (failure to use all the points on the rating scale, not discriminating among competencies). Straight line scoring occurs when the rater ignores the traits to be rated and treats all traits as if they were one. Thus, ratings tend to be repetitious and inflate estimates of reliability. Better training of faculty tasked with the assessments may help to reduce some undesirable rater effects [8].

Rotation directors typically work directly with a resident, and also obtain input from the other faculty when completing the end-of-rotation evaluations. However, straight line scoring indicates that individualized assessment may not be occurring. This could be due to several reasons including lack of faculty expertise in assessment, lack of valid and reliable methods for assessment of specific milestones, and lack of resources to actually complete the assessment due to lack of protected time in busy clinical rotations. Overcoming these barriers is important to optimize graduate medical education. In addition, the ACGME Review Committees plan to examine milestone performance data for each program’s residents as one element in the Next Accreditation System.

Using the results of this study to make enhancements to improve the resident evaluation process yields several potential interventions. One possibility is to reduce the number of subcompetencies expected to be evaluated in each rotation from the current average of 18 to a lower more manageable realistic number that is best addressed by the nature of that specific rotation. This would ideally allow the rotation director to focus on properly evaluating a few subcompetencies, but at the trade-off of fewer repeat evaluations of the subcompetency as the resident progresses. Reducing the number of evaluations that have straight line scoring would indicate more robust resident assessment.

Another mechanism to improve the evaluation process is to have faculty receive more extensive instructions on the assessment of residents, the meaning of the subcompetencies, and the scaling of the milestones. For this study, training of rotation directors only occurred once but based on these results training will occur annually. Ideally, multiple assessments (e.g., test scores, conference attendance, direct observations) are combined to inform decisions on different milestones [9]. There is turnover among the faculty so new rotation directors need to be educated and mentored. This study also found a delay in that 37% of evaluations were entered into the system for the resident to see more than one month after rotation end. This likely reduces the impact on the resident to be able to modify any behavior. As a result, it may be worthwhile to examine different types of information to provide evaluators (e.g., a real-time dashboard with summary data on their timeliness of evaluations submitted as well as the frequency of straight line scoring) that might help change behavior and improve the information residents receive on the evaluations.

Another consideration anecdotally brought up by faculty and residents is to change the evaluation scale so that the milestone level does not directly correspond with the post-graduate year. It may be too easy to fall in the mindset of postgraduate year and milestone level being tied as the same regardless of residents’ abilities in the specific subcompetencies. Since faculty may be thinking of the resident’s postgraduate year when completing the evaluation, they are in fact confirming that the resident is at appropriate level.

Multiple studies do exist comparing faculty-based milestone evaluations with resident’s self-assessments on those same milestones [10-12]. For example, in comparing 20 general surgery residents’ self-evaluations with the Clinical Competence Committee evaluation of their performance, most self-assessments were within half a level of the committee’s report [10]. In a study of all US Internal Medicine Residents, milestone-based rating by the faculty correlated with the prior non-developmentally based (unsatisfactory, satisfactory, superior) resident evaluation rating scale [13].

A few of the residents on some of the rotations had subcompetencies below a Level 4 within the last three months of training. A national study of all United States Internal Medicine residents, similarly found a fraction of graduating residents who had competency ratings below Level 4 [14]. In a Pediatric Residency, variation in milestone scores decreased over time and by graduation, almost all residents scored did not have a Level 4 score or greater in all subcompetencies [15].

Several limitations to this study exist because descriptive research focuses on details or characteristics of a population (our small sample size) without making inferences beyond those who are studied [16]. For example, there may be confounding issues related to rotation evaluation scores such as faculty being reluctant to assign low scores to residents because of fear that the resident may retaliate by judging the faculty poorly on the resident evaluation of the attending. Also, raters may not assess a resident as performing poorly for not wanting to delay advancement of the resident or fear of the repercussions of adding extra residency time [17], and the risk of other rater biases including gender bias [18,19]. These dimensions and further investigation as why straight line scoring is occurring which could be done qualitatively such as by interviewing the faculty rotation directors was not undertaken in this study.

Further studies can examine what interventions can better provide meaningful data for Clinical Competency Committees, support self-directed assessments, facilitate resident feedback, and allow specialty Review Committees to monitor and help programs improve.

## Conclusions

The milestones provide a framework for the assessment of the development of the resident from novice to expert. Descriptive analysis of a residency program’s end-of-rotation milestone evaluations may yield data to target improvement efforts so that the competency-based evaluation can attain its intended goals.

## References

[1] Nasca TJ, Philibert I, Brigham T, Flynn TC (2012). The next GME accreditation system — rationale and benefits. N Engl J Med.

[2] Norcini J, Burch V (2007). Workplace-based assessment as an educational tool: AMEE guide no 31. Med Teach.

[3] Hawkins RE, Holmboe ES, Durning SJ (2008). Practical Guide to the Evaluation of Clinical Competence. https://www.elsevier.com/books/practical-guide-to-the-evaluation-of-clinical-competence/holmboe/978-0-323-44734-8.

[4] (2018). Anesthesiology Milestones ACGME Report Worksheet. https://www.acgme.org/Portals/0/PDFs/Milestones/AnesthesiologyMilestones.pdf.

[5] Ross FJ, Metro DG, Beaman ST (2016). A first look at the Accreditation Council for Graduate Medical Education anesthesiology milestones: implementation of self-evaluation in a large residency program. J Clin Anesth.

[6] Park YS, Zar FA, Norcini JJ, Tekian A (2016). Competency evaluations in the next accreditation system: contributing to guidelines and implications. Teach Learn Med.

[7] Peabody MR, O'Neill TR, Peterson LE (2017). Examining the functioning and reliability of the family medicine milestones. J Grad Med Educ.

[8] Downing S, Yudkowsky R (2009). Assessment in Health Professions Education. https://www.routledge.com/Assessment-in-Health-Professions-Education/Downing-Yudkowsky/p/book/9780805861280.

[9] Friedman KA, Raimo J, Spielmann K, Chaudhry S (2016). Resident dashboards: helping your clinical competency committee visualize trainees’ performance indicators. Med Educ Online.

[10] Watson RS, Borgert AH, O’Heron CT (2017). A multicenter prospective comparison of the Accreditation Council for Graduate Medical Education milestones: clinical competency committee vs resident self-assessment. J Surg Educ.

[11] Lyle B, Borgert AJ, Kallies KJ, Jarman BT (2016). Do attending surgeons and residents see eye to eye? An evaluation of the Accreditation Council for Graduate Medical Education milestones in general surgery residency. J Surg Educ.

[12] Yao A, Massenburg BB, Silver L, Taub PJ (2017). Initial comparison of resident and attending milestones evaluations in plastic surgery. J Surg Educ.

[13] Hauer KE, Vandergrift J, Hess B (2016). Correlations between ratings on the resident evaluation summary and the internal medicine milestones and associations with ABIM certification examination scores among US internal medicine residents. JAMA.

[14] Hauer KE, Clauser J, Lipner RS (2016). The internal medicine reporting milestones cross-sectional description of initial implementation in U.S. residency programs. Ann Intern Med.

[15] Li ST, Tancredi DJ, Schwartz A (2017). Competent for unsupervised practice use of pediatric residency training milestones to assess readiness. Acad Med.

[16] Newton Suter W (2012). Introduction to Educational Research: A Critical Thinking Approach.

[17] Kogan JR, Conforti L, Bernabeo E, Iobst W, Holmboe E (2011). Opening the black box of clinical skills assessment via observation: a conceptual model. Med Educ.

[18] Dayal A, O'Connor DM, Qadri U, Arora VM (2017). Comparison of male vs female resident milestone evaluations by faculty during emergency medicine residency training. JAMA Intern Med.

[19] Schwind CJ, Williams RG, Boehler ML, Dunnington GL (2004). Do individual attendings' post-rotation performance ratings detect residents' clinical performance deficiencies?. Acad Med.

